# Inorganic nitrate ingestion improves vascular compliance but does not alter flow-mediated dilatation in healthy volunteers

**DOI:** 10.1016/j.niox.2012.01.004

**Published:** 2012-05-15

**Authors:** M. Bahra, V. Kapil, V. Pearl, S. Ghosh, A. Ahluwalia

**Affiliations:** William Harvey Research Institute, Centre for Clinical Pharmacology, Barts & the London School of Medicine and Dentistry, Queen Mary University of London, London, United Kingdom

**Keywords:** Nitrite, Endothelium, Human, Blood flow

## Abstract

Ingestion of inorganic nitrate elevates blood and tissue levels of nitrite via bioconversion in the entero-salivary circulation. Nitrite is converted to NO in the circulation, and it is this phenomenon that is thought to underlie the beneficial effects of inorganic nitrate in humans. Our previous studies have demonstrated that oral ingestion of inorganic nitrate decreases blood pressure and inhibits the transient endothelial dysfunction caused by ischaemia–reperfusion injury in healthy volunteers. However, whether inorganic nitrate might improve endothelial function *per se* in the absence of a pathogenic stimulus and whether this might contribute to the blood pressure lowering effects is yet unknown. We conducted a randomised, double-blind, crossover study in 14 healthy volunteers to determine the effects of oral inorganic nitrate (8 mmol KNO_3_) vs. placebo (8 mmol KCl) on endothelial function, measured by flow-mediated dilatation (FMD) of the brachial artery, prior to and 3 h following capsule ingestion. In addition, blood pressure (BP) was measured and aortic pulse wave velocity (aPWV) determined. Finally, blood, saliva and urine samples were collected for chemiluminescence analysis of [nitrite] and [nitrate] prior to and 3 h following interventions. Inorganic nitrate supplementation had no effect on endothelial function in healthy volunteers (6.9 ± 1.1% pre- to 7.1 ± 1.1% post-KNO_3_). Despite this, there was a significant elevation of plasma [nitrite] (0.4 ± 0.1 μM pre- to 0.7 ± 0.2 μM post-KNO_3_, *p* < 0.001). In addition these changes in [nitrite] were associated with a decrease in systolic BP (116.9 ± 3.8 mm Hg pre- vs. 112.1 ± 3.4 mm Hg post-KNO_3_, *p* < 0.05) and aPWV (6.5 ± 0.1 m/s pre- to 6.2 ± 0.1 post-KNO_3_, *p* < 0.01). In contrast KCl capsules had no effect on any of the parameters measured. These findings demonstrate that although inorganic nitrate ingestion does not alter endothelial function *per se*, it does appear to improve blood flow, in combination with a reduction in blood pressure. It is likely that these changes are due to the intra-vascular production of NO.

## Highlights

► Inorganic nitrate supplementation does not alter endothelial function in healthy volunteers. ► Despite this, there was a reduction in blood pressure and improvement in arterial compliance. ► Improvements in vascular function are likely to contribute to the beneficial effects of inorganic nitrate on blood pressure.

## Introduction

The healthy endothelium plays an essential role in regulating vascular homeostasis by endowing the blood vessel wall with a vasodilator, anti-platelet, anti-atherogenic and anti-proliferative phenotype [1,2]. An important mediator of this beneficial influence of the endothelium is nitric oxide (NO) which is generated tonically by endothelial NO synthase (eNOS) [3,4]. Importantly, in patients with cardiovascular risk factors these beneficial effects of the endothelium are impaired [5]; a phenomenon termed ‘endothelial dysfunction’. This dysfunction, at least in part, has been attributed to a reduced synthesis and bioavailability of endothelium-derived NO [6–8] that has been evidenced by the measurement of reductions in the levels of NO metabolites and via the clinical assessment of reduced endothelial function by measuring flow-mediated dilatation (FMD), an endothelium-dependent vasodilatation response known to be mediated by NO [9].

Endothelial dysfunction often precedes symptomatic cardiovascular disease [5], offering potential for measurement as a biomarker [10], but is thought also to be a key pathogenic event involved in the development of disease [11]. Clinical evidence supports the view that the magnitude of the FMD response is also predictive not only of the occurrence of an event in both low- and high-risk individuals [12–14] but also the severity of cardiovascular disease [15,16]. Therefore, identifying novel approaches that might improve or recover NO-dependent endothelial responses in individuals with cardiovascular disease may offer potential therapeutic strategies worth exploring.

Recently, there has been a growing appreciation that the inorganic nitrate $\left({\text{NO}}_{3}^{-}\right)$ anion, either generated *in vivo* as an oxidative metabolite of NO itself [17] or ingested through the diet (predominant dietary sources being green leafy vegetables) [18] might offer a source of NO in the body that is independent of the conventional NO synthesis pathways [19,20]. In the body nitrate anion is concentrated in the salivary glands and secreted within the saliva into the oral cavity [21]. Here, this nitrate-rich saliva comes into close contact with facultative anaerobes, on the dorsal surface of the tongue, that reduce nitrate to nitrite [22]. This nitrite is then swallowed, absorbed and distributed within the vasculature, raising systemic nitrite levels [23,24]; a circuit often described as the entero-salivary circuit [23,25]. Within the circulation, nitrite is then reduced to bioactive NO by one of a number of potential enzymatic and non-enzymatic processes [26], a phenomenon supported by demonstration of associated elevations of plasma cyclic guanosine monophosphate (cGMP) levels, thought to be the most sensitive indicator of endogenous NO generation [27]. Importantly, our previous studies have demonstrated that ingestion of inorganic nitrate lowers blood pressure (BP) and also prevents the endothelial dysfunction caused by an ischaemia–reperfusion injury in the forearm of healthy volunteers [23,24]. However, whether these effects of inorganic nitrate relate simply to provision of NO or whether alteration of endothelial function *per se* (as has been evidenced for other albeit unrelated dietary-based interventions [28,29]) might underlie these effects is yet unknown and an issue we investigated in the study described herein.

## Materials and methods

### Volunteers

The studies were granted full ethical approval by the local research ethics committee. All participants gave informed consent after satisfying the inclusion criteria: healthy male or female adults aged between 18 and 45 years; systolic blood pressure (SBP) <140 mm Hg and diastolic blood pressure (DBP) <90 mm Hg; body mass index (BMI) 18–30 kg/m^2^, non-smoker and no systemic medication (except the oral contraceptive pill). Volunteers were instructed to eat a low nitrate diet (i.e. no green leafy vegetables or cured meats) and avoid strenuous exercise the day before study visits. In addition, volunteers were fasted overnight before all study visits.

### Study protocol

Power analyses, based upon our previous findings [23,24] indicated that to obtain significant elevation (2-fold) of circulating nitrite levels an *n* of 12 would be sufficient using an *α* = 0.05, *β* = 0.90 and a standard deviation of 0.37 μM. We conducted a randomised, placebo-controlled, double blind, cross-over, clinical study in 14 healthy volunteers (additional two to account for potential dropouts), who received either 8 mmol potassium nitrate (KNO_3_, Martindale Pharmaceuticals, Ipswich, UK) or matched potassium chloride (KCl, Martindale Pharmaceuticals, Ipswich, UK) placebo control, and completed the cross-over limb between 7 and 28 d later. Interventions were taken with 2 slices of dry wholemeal toast and 250 ml low-nitrate containing water (Zepbrook Ltd., London, UK). BP was measured and urine, saliva and plasma samples were collected for analysis of [nitrate] and [nitrite] (collectively termed ‘NO*_x_*’) by ozone chemiluminescence. FMD of the brachial artery was measured using ultrasound and arterial compliance by measurement of aortic pulse wave velocity (aPWV). All studies took place in a quiet, temperature controlled (24–26 °C) environment. All measures were performed at baseline and at 3 h post intervention. This 3 h time point was chosen since our previous findings have demonstrated that circulating levels of nitrite and the associated bioactivity (i.e. decrease in BP, prevention of ischaemia–reperfusion induced endothelial dysfunction, etc.) peak at this time following nitrate ingestion [23,24].

### Study techniques

#### Blood pressure measurement

All BP and heart rate (HR) measurements were performed in triplicate in the seated position using an Omron 705IT according to established guidelines [30]. Both investigators and participants were blinded to the readings by means of laminated coverings for the machine and printer. For the purpose of analysis, the means of the 2nd and 3rd readings were used.

#### FMD measurement

Endothelial function was assessed by measuring brachial artery diameter in response to reactive hyperaemia as previously described [23,31]. Briefly, a B-mode scan of the brachial artery was obtained in longitudinal section above the antecubital fossa using a 7.0 MHz linear array transducer and a standard Acuson Aspen system (Acuson, Mountain View, UK). Arterial diameter over a 1–2 cm section was determined for each image with the use of automatic edge-detection software (Vascular Research Tools, Medical Imaging Applications LLC Iowa City, USA) and synchronised by 3-lead electrocardiography to the *R*-wave of the QRS complex. Blood flow was altered in the brachial artery by a 7 cm-wide BP cuff placed immediately below the antecubital fossa around the forearm. After 1 min of baseline flow, the cuff was inflated to 300 mm Hg for 5 min and released, resulting in a brief episode of reactive hyperaemia and changes in brachial artery diameter in response to blood flow were assessed for an additional 5 min. Brachial artery diameter and brachial artery dilation, expressed as percentage increase from baseline diameter, were determined using the software Vascular Analysis Tools (Medical Imaging Applications, LLC Iowa City, USA).

#### aPWV measurement

A Vicorder device (Skidmore Medical Ltd., Bristol, UK) was used to simultaneously record the pulse wave from the carotid and femoral arteries using an oscillometric method [32]. A small, inflatable neck pad is placed directly over a single carotid artery and secured around the neck by a Velcro tab and a cuff is placed around the subject’s ipsilateral upper thigh. Both carotid and femoral cuffs are inflated automatically to 65 mm Hg and the corresponding oscillometric signal from each cuff is digitally analysed to extract the pulse-time delay. The distance between the sternal notch and the thigh cuff is measured and used as a standard estimate for the aortic length. From these measurements aortic pulse wave velocity (aPWV) can be derived as aPWV = aortic distance/pulse time delay.

#### Blood sampling

Blood was sampled into separate pre-chilled EDTA tubes for NO*_x_*, through a 21-gauge butterfly needle inserted into the antecubital vein. Blood samples were immediately centrifuged (1300*g*, 4 °C, 10 min), plasma was separated and deproteinated by filtration (centrifugation 14000*g*, 4 °C, 60 min) using *Vivaspin 500*, 3 kDa filters (Sartorius Biotech, Aubagne, France) and the filtrate stored at −80 °C, until analysis by ozone chemiluminescence.

#### Urine and saliva sampling

Mid-stream urine samples were collected into sterile containers and an aliquot stored at −80 °C until analysis at a later date. Unstimulated saliva was collected into sterile eppendorfs and centrifuged (14000*g*, 4 °C, 10 min) and the supernatant was collected and stored at −80 °C until analysis at a later date.

#### Measurement of plasma [NO*_x_*]

[NO*_x_*] was measured by ozone-based chemiluminescence as previously described [17]. Briefly, to determine total [NO*_x_*], samples were added to 0.1 M vanadium (III) chloride in 1 M hydrochloric acid refluxing at 95 °C under nitrogen. [Nitrite] was determined by addition of samples to 0.09 M potassium iodide in glacial acetic acid under nitrogen at room temperature. [Nitrate] was calculated by subtraction of the [nitrite] from [NO*_x_*].

### Statistical analysis

The data were analysed using Graphpad Prism software version 5. All data are expressed as a mean ± SEM. For FMD and aPWV responses, data were compared using paired Student *t*-tests. Plasma, urine and saliva [NO*_x_*] were analysed using one-way ANOVA and Bonferroni post hoc tests for comparison of individual treatments. A *p*-value <0.05 was considered to be significant for all analyses.

## Results

All interventions were tolerated without adverse effects. Volunteer baseline demographics are presented in Table 1 and demonstrate no significant differences in baseline characteristics or baseline [NO*_x_*] between the two limbs of the study. For one volunteer, FMD could not be reliably measured due to technical difficulties.

### Inorganic nitrate supplementation raised [NO*_x_*]

**Fig. 1:**
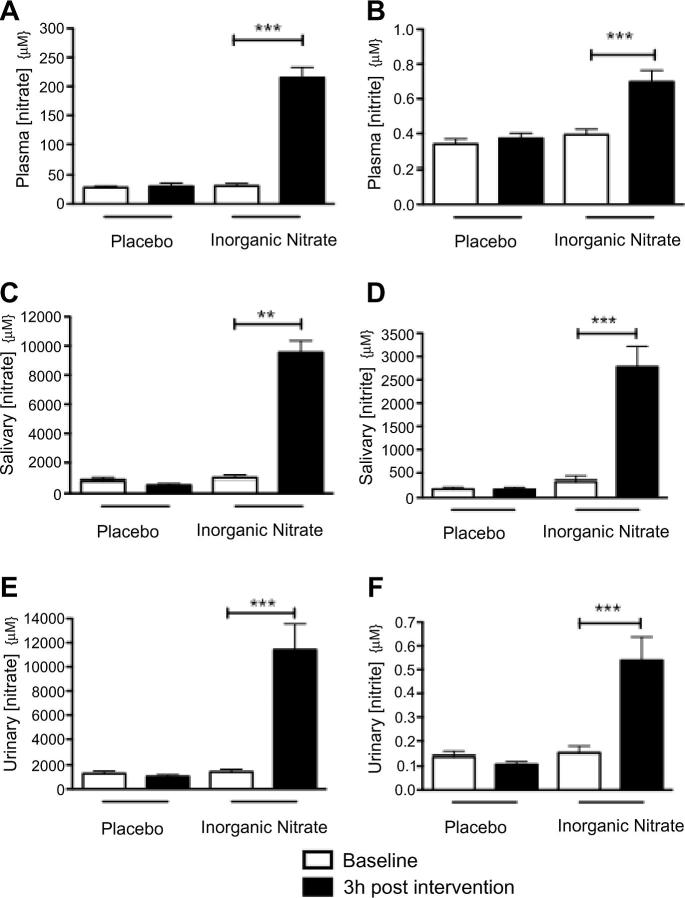
Inorganic nitrate supplementation elevates NO*_x_* in healthy volunteers. The effects of inorganic nitrate supplementation (KNO_3_, 8 mmol) or placebo (KCl, 8 mmol) on circulating plasma (a) [nitrate] and (b) [nitrite]; salivary (c) [nitrate] and (d) [nitrite]; and urinary (e) [nitrate] and (f) [nitrite]. Significance shown for Bonferroni post hoc tests between groups as ^∗∗^*p* < 0.01 and ^∗∗∗^*p* *<* 0.001 following 1-way ANOVA. Data are expressed as mean ± SEM (*n* = 14).

At 3 h following ingestion of inorganic nitrate there was a significant elevation in plasma, salivary and urinary [nitrate] and [nitrite] (Fig. 1). There were no changes in plasma, salivary or urinary [NO*_x_*] after placebo (Fig. 1) (*n* = 14 for all).

### Inorganic nitrate supplementation does not alter endothelial function

**Fig. 2:**
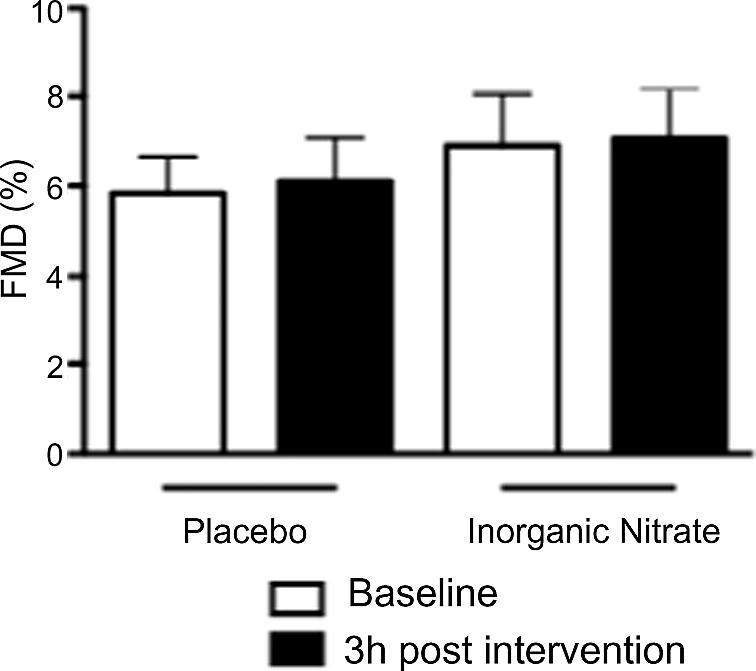
Inorganic nitrate does not alter endothelial function in healthy volunteers. The effects of inorganic nitrate supplementation (KNO_3_, 8 mmol) or placebo (KCl, 8 mmol) on FMD. No significance found for comparisons between groups using Bonferroni post hoc tests, following 1-way ANOVA. Data are expressed as mean ± SEM (*n* = 13).

There was no significant difference in baseline brachial artery diameter between the groups, with a mean diameter of 3.4 ± 0.2 mm (Table 2). In addition, there was no evidence of change in the FMD response after either inorganic nitrate supplementation or placebo (*n* = 13 for both) (Fig. 2).

### Inorganic nitrate supplementation reduces BP and aPWV

**Fig. 3:**
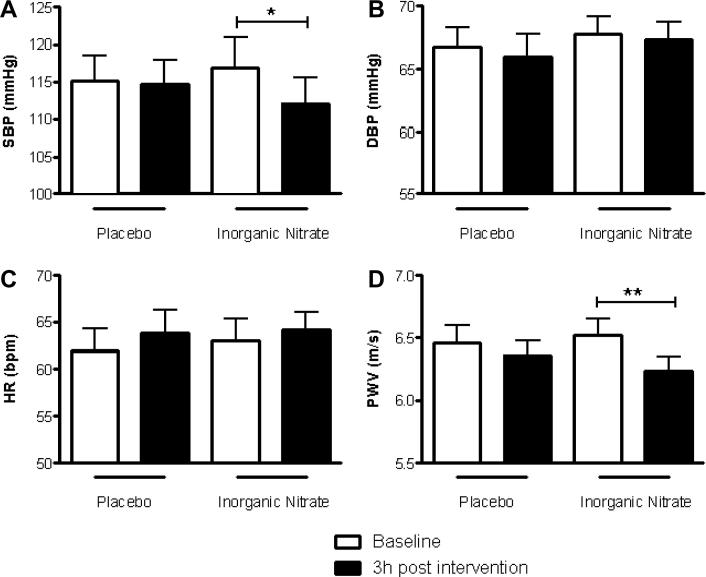
Inorganic nitrate reduces SBP and aPWV in healthy volunteers. The effects of inorganic nitrate supplementation (KNO_3_, 8 mmol) or placebo (KCl, 8 mmol) on (a) SBP, (b) DBP, (c) HR and (d) aPWV. Significance shown for Bonferroni post hoc tests between groups as ^∗^*p* *< *0.05, ^∗∗^*p* < 0.01 following 1-way ANOVA. Data are expressed as mean ± SEM (*n* = 14).

There was a significant reduction in systolic BP (SBP) after inorganic nitrate supplementation (ΔSBP = −4.9 ± 1.4 mm Hg, *p* < 0.05, Fig. 3a) that was not apparent after placebo (ΔSBP = −0.4 ± 1.3 mm Hg; Fig. 3a). There were no significant changes in diastolic BP (DBP) or heart rate (HR) after inorganic nitrate supplementation or placebo (Fig. 3b and c). Similarly to SBP, aPWV was significantly reduced after inorganic nitrate supplementation but unchanged after placebo (Fig. 3d).

## Discussion

Fruit and vegetable rich diets are associated with reduced risk of cardiovascular disease [33,34]. Prospective clinical studies investigating the potential of dietary anti-oxidants in protecting against cardiovascular disease have failed to reproduce the same beneficial effects [35–37]. Such observations have stimulated the search for other possible candidates that might underlie the benefits of fruit and vegetable rich diets. This search has lead some to suggest that dietary (inorganic) nitrate may be responsible [18,20,38]. Herein, we demonstrate that whilst inorganic nitrate supplementation does not alter endothelial function *per se* that it does improve vascular compliance; an effect that is also associated with a decrease in BP, in healthy volunteers. We suggest that these effects are likely to play a role in the protective effects attributed to healthy diets.

The non-invasive assessment of endothelial function has proven to be a useful surrogate marker with which to assess both general cardiovascular health and risk of cardiovascular events [7]; in particular, the measurement of ultrasound FMD [12,39]. Our previous studies have shown that dietary nitrate and inorganic nitrate salts are able to prevent the decreases in FMD, as a reflection of endothelial dysfunction, induced by ischaemia–reperfusion injury in healthy volunteers [23,24]. However, whether these effects related to prevention of the damage caused by the pathogenic insult or whether the dietary nitrate intervention improved endothelial function *per se* were not determined. Here, we show in healthy volunteers that despite elevations in systemic [nitrite] following ingestion of an 8 mmol dose of KNO_3_ salt, there was no evidence of alterations in endothelial function. Previous studies have shown that certain acute interventions can enhance FMD responses in individuals classified as healthy volunteers. For instance following an acute intervention of flavonoid-rich green tea [29] or dark chocolate [28] FMD responses in healthy volunteers were enhanced; effects proposed to contribute substantially to the apparent protective effects of these dietary substances against cardiovascular disease [40,41]. Such observations indicate that the absence of an effect of the intervention in this study was not simply due to the fact that FMD responses cannot be enhanced in healthy volunteers. Interestingly, enhanced endothelial NO synthesis or bioavailability has been implicated as underlying the improvements in FMD in response to a number of distinct dietary constituents [42,43]. However, studies attempting to directly modulate endogenous NO-generating pathways, such as acute l-arginine (eNOS substrate) supplementation have failed to alter FMD responses in healthy volunteers [44].

This lack of effect of nitrate supplementation on endothelial function was not related to a lack of bioactivity since, as in other studies [23–25], inorganic nitrate supplementation reduced SBP by ∼4.9 mm Hg, 3 h post intervention; an effect that is directly correlated with elevations in circulating nitrite concentrations [23,24]. In this study we chose to use a dose of 8 mmol KNO_3_ to reflect the dose that one might consume in a vegetable-rich meal [18,38,45]. In addition this dose was chosen since it is an intermediate dose between 4 and 12 mmol KNO_3_ which we have previously shown to cause dose-dependent rises in circulating nitrite levels with dose-dependent reductions in SBP in healthy volunteers (by ∼2 and ∼5.6 mm Hg, respectively) [24]. These BP-lowering effects of 8 mmol KNO_3_ lend further support for a dose-response relationship of inorganic nitrate supplementation with respect to BP lowering.

Our studies also demonstrated an additional modest yet significant reduction in aPWV, which is a measure of arterial compliance. Arterial compliance in large, elastic arteries is determined by both the distending pressure and the intrinsic wall properties [46]. It is unlikely that an acute administration of inorganic nitrate alters structural properties and therefore the increase in compliance is most likely a consequence of alterations in the distending pressure in the aorta. Previous studies have shown similar findings where acute alterations of endogenous NO levels alter BP and vascular compliance concomitantly. Chowienczyk and colleagues demonstrated that administration of organic nitrovasodilators produced a very rapid reduction in BP in healthy volunteers associated with an increased arterial compliance [47]. Similarly, acute inhibition of NO synthesis using the NOS inhibitor l-NMMA produced local reductions in arterial compliance [48].

Arterial compliance as measured by aPWV is a strong predictor of future cardiovascular mortality [49] and has been adopted as an important measure of cardiovascular risk prediction schemes [50]. Whether the effects of acute inorganic nitrate supplementation on arterial compliance might be sustained with chronic ingestion and whether such an effect might alter hypertension and cardiovascular risk are unknown. However, our research provides further support for encouraging such investigations.

### Limitations

Although our study had a small sample size, we were adequately powered to detect changes in circulating plasma [nitrite] and BP changes after inorganic nitrate supplementation. Similarly, we had chosen to study volunteers at 3 h post intervention based upon our previous studies demonstrating peak [nitrite] elevations and BP responses after nitrate supplementation at ∼3 h post intervention and importantly protection against ischaemia–reperfusion-induced endothelial dysfunction [23,24]. However, it is possible that changes in basal FMD responses occurred at earlier or indeed later time-points.

## Conclusion

These findings demonstrate that although inorganic nitrate ingestion does not alter endothelial function *per se*, it does improve blood flow, in combination with a reduction in blood pressure. It is likely that these changes are due to the production of NO that likely directly relaxes vascular smooth muscle and therefore decreases aPWV.

## Figures and Tables

**Table 1:** Baseline demographic, haemodynamic and analytical parameters.

Characteristic	Placebo limb	Inorganic nitrate limb	Significance
Subjects (*n*)	14		
Age (years)	27.9 ± 1.8		
BMI (kg/m^2^)	24.5 ± 0.9		
Baseline SBP (mm Hg)	115.1 ± 3.4	116.9 ± 3.8	*p* = 0.37
Baseline DBP (mm Hg)	66.8 ± 1.5	67.7 ± 1.4	*p* = 0.54
Plasma $\left[{\text{NO}}_{3}^{-}\right]$ (μM)	28.5 ± 2.4	31.1 ± 4.2	*p* = 0.54
Plasma $\left[{\text{NO}}_{2}^{-}\right]$ (μM)	0.39 ± 0.03	0.34 ± 0.03	*p* = 0.20

Significance shown in the last column for paired Student’s *t*-test. Data are shown as mean ± SEM values (*n* = 14).

**Table 2:** Brachial artery diameter across all 4 visits.

	Placebo limb		Inorganic nitrate limb		
Characteristic	Pre-	3 h post	Pre-	3 h post	Significance
Baseline brachial artery diameter (mm)	3.3 ± 0.2	3.4 ± 0.2	3.4 ± 0.2	3.4 ± 0.2	*p* = NS for all comparisons

No significance found with Bonferroni post hoc tests following 1-way ANOVA (*n* = 13).
